# Effect of Pre-Strain Induced Microstructure Evolution on Hydrogen Embrittlement Resistance of a CoCrNi Medium-Entropy Alloy

**DOI:** 10.3390/ma18214915

**Published:** 2025-10-27

**Authors:** Zening Wang, Sirui Jing, Yu Yan

**Affiliations:** Beijing Advanced Innovation Center for Materials Genome Engineering, Corrosion and Protection Center, Institute for Advanced Materials and Technology, University of Science and Technology Beijing, Beijing 100083, China; wangzeningning@163.com (Z.W.); jing_sr@163.com (S.J.)

**Keywords:** medium-entropy alloy, hydrogen embrittlement, fracture, deformation twins

## Abstract

The effect of pre-strain-induced microstructural evolution on the hydrogen embrittlement (HE) resistance of an equiatomic CoCrNi medium-entropy alloy was systematically investigated by mechanical property testing, scanning electron microscopy (SEM), and electron backscatter diffraction (EBSD) characterization. Three pre-strain levels (0%, 30%, and 50%) were applied to produce distinct microstructures: dislocation-free and twin-free (P0), high dislocation density with few deformation twins (P30), and high densities of both dislocations and deformation twins (P50). Mechanical tests combined with hydrogen charging revealed that the P50 specimen exhibited the highest yield strength (1163.88 MPa) and the lowest HE-induced elongation loss (2.74%), indicating an improvement in HE resistance. By using SEM, detailed observations of the fracture morphology and crack propagation paths revealed that deformation twins can effectively reduce stress concentration, delay the nucleation and propagation rates of cracks, and suppress brittle intergranular fracture, thereby improving mechanical properties and resistance to hydrogen embrittlement. A detailed analysis was conducted of the HE resistance mechanism associated with the influence of deformation twins on hydrogen transport and distribution.

## 1. Introduction

Hydrogen embrittlement (HE) is a prevalent failure phenomenon in metallic structural materials. Most engineering alloys are susceptible to severe degradation of mechanical properties when exposed to hydrogen-containing environments, posing a significant challenge to the application of structural materials in H-abundant harsh environments [1,2]. To develop structural materials with both outstanding mechanical properties and HE resistance, multi-principal element alloys (MPEAs) have attracted increasing research interest. MPEAs contain at least three principal elements with concentrations ranging from 5 to 35 at.% and exhibit typical high-entropy alloy characteristics, including simple microstructures, severe lattice distortion, and highly tunable properties. Representative alloys include FeMnCoCrNi, FeMnCoCr, and CoCrNi alloys composed of representative 3D transition elements have demonstrated excellent physical properties [3], enhanced low-temperature work hardening capacity [4] and exceptional strength–ductility balance [5]. Thus, it is crucial to investigate the HE resistance and mechanisms of these alloys, based on their excellent physical and mechanical properties as well as their potential applications [6,7].

The CoCrNi medium-entropy alloy (MEA) has attracted substantial research attention due to the exceptional thermodynamic stability of its single-phase face-centered cubic (FCC) structure. This stable crystallographic configuration gives rise to an outstanding combination of properties, including superior mechanical performance characterized by high strength and considerable ductility as reported in reference [8], complemented by significant resistance to high-temperature oxidation [9] and exceptional corrosion resistance [10]. Such a comprehensive set of advantageous characteristics establishes the CoCrNi MEA as a particularly promising candidate material for advanced engineering applications under extreme service conditions. Although a growing number of scholars have investigated the HE resistance of the CoCrNi MEAs and proposed various microscopic mechanisms to explain it from different perspectives [11,12,13,14], while these studies remain insufficient. Soundararajan et al. found that the equi-molar CoCrNi MEA exhibited excellent HE resistance after electrochemical hydrogen charging, which was attributed to its low hydrogen diffusion rate and the hydrogen-induced deformation twins that effectively hindered the propagation of hydrogen-induced cracks [12]. Yi et al. found that the CoCrNi alloy exhibited a significant reduction in elongation via gaseous hydrogen charging. Their study revealed that its low resistance to HE primarily originated from the hydrogen-enhanced decohesion (HEDE) mechanism. However, the Mo-alloyed (CoCrNi)_97_Mo_3_ MEA exhibited enhanced HE resistance due to the Mo-promoted twinning-dominated deformation mechanism, which suppresses hydrogen redistribution via dislocation motion and inhibits hydrogen-induced grain boundary (GB) decohesion [15]. Fu et al. demonstrated that hydrogen has contradictory effects on the mechanical properties of equiatomic CoCrNi MEA. Hydrogen weakens GBs causing intergranular cracking, yet promotes deformation twinning that enhances crack propagation resistance [16].

In general, as an effective method for strengthening face-centered cubic (FCC) matrices, pre-straining has been widely adopted [17,18], primarily implemented through processes such as rolling or severe plastic deformation (SPD). Under certain conditions, pre-strain induced twins can promote ductile fracture and mitigate HE [19]. However, these twins may also act as pathways for crack propagation, thereby exacerbating HE [20]. The influence of pre-strain generated twins on HE depends on multiple factors, including the material’s intrinsic deformation mechanism [21], twin size characteristics [22], and dislocation density/configuration [23]. Notably, research on CoCrNi equiatomic MEA has shown that CoCrNi MEA features severe lattice distortion and low stacking fault energy, which to some extent hinders dislocation glide during deformation, making it more prone to the formation of nano-deformation twins [4,24,25]. However, current research on the interactions between these deformation twins and hydrogen, as well as their effects on mechanical properties, remains highly inadequate.

In this study, the effect of varying pre-strain levels on the microstructure was investigated, introducing dislocations and deformation twins to examine hydrogen behavior under different microstructural conditions and its impact on mechanical properties. Fracture surfaces were characterized in detail using SEM to analyze crack morphology and identify fracture characteristics, thereby elucidating the intrinsic correlations and microscopic mechanisms among pre-strain-induced microstructure, hydrogen behavior, and fracture response. Electrochemical hydrogen charging was employed for hydrogen pre-charging, and mechanical properties were evaluated at a strain rate of 5 × 10^−5^ s^−1^.

## 2. Materials and Methods

A 2 kg equiatomic CoCrNi MEA ingot was prepared through vacuum floating melting using pure elements (>99.9%) supplied by Beijing Yanbang New Material Technology Co., Ltd. (Beijing, China) as raw materials. After the ingot was obtained, it was hot rolled at 1100 °C to a thickness reduction ratio of 50%. Then, the sample was cold rolled to a thickness reduction ratio of 40%, and a final plate with a thickness of 1.5 mm was obtained. Subsequently, the plate was annealed at 1100 °C for 30 min to obtain a completely recrystallized structure; then, the plate was water quenched to room temperature. All heat treatments were conducted in a protected argon environment.

Tensile test specimens were cut from the plate after heat treatment. The size of the tensile test specimen was 60 mm × 5 mm × 1.5 mm (the length direction is parallel to the rolling direction), and the size of gauge was 15 mm × 5 mm × 1.5 mm. The tensile test specimens were pre-strained at a strain rate of 1 × 10^−3^ s^−1^. Three groups of samples were prepared with engineering strains of 0%, 30%, and 50%, to obtain different microstructures, and these three sets were denoted as P0, P30, and P50 in this work, respectively. To ensure consistency, all tensile test specimens were polished using silicon carbide paper to 5000 mesh. The geometry of the gauge area in the tensile test specimens need to be controlled because after pre-strain, the geometric size of the gauge changes, and the cross-sectional area became smaller. For the P50 sample, the cross-sectional area changed from 5 mm × 1.5 mm to 4.2 mm × 1.2 mm. Thus, to ensure consistent conditions for mechanical testing, the cross-sectional dimensions of both P0 and P30 samples were carefully controlled. To this end, they were processed by grinding and mechanical polishing to achieve a uniform cross-sectional area of 4.2 mm × 1.2 mm, while the gauge length was maintained at a constant value of 15 mm.

The electrolyte consisted of 0.5 mol L^−1^ H_2_SO_4_ + 2 g L^−1^ CH_4_N_2_S solution, and a DC power supply was used for hydrogen charging. Hydrogen charging continued for 48 h at a hydrogen charging current density of 50 mA cm^−2^. The slow strain rate tensile tests were conducted at room temperature using an Instron 5967 universal testing machine (Instron, Norwood, MA, USA). To ensure consistency and comparability, all specimens were meticulously prepared and clamped according to identical geometric standards. The tests were performed under a constant strain rate control mode at 5 × 10^−5^ s^−1^ until fracture. This specific, deliberately chosen strain rate is sufficiently slow to facilitate the dynamic interaction between hydrogen diffusion and dislocations, which is critical for effectively revealing the material’s susceptibility to hydrogen embrittlement. HE sensitivity was assessed by total elongation loss (*EI*_loss_), defined as follows:

(1) $${\mathrm{EI}}_{\mathrm{loss}}=\;\frac{{\mathrm{EI}}_{\mathrm{free}\;}-\;{\mathrm{EI}}_{H\;}}{{\mathrm{EI}}_{\mathrm{free}\;}}\;\times \;100\%$$

where ${\mathrm{EI}}_{\mathrm{free}\;}$ is the tensile elongation of the H-free tensile test specimens, and ${\mathrm{EI}}_{H\;}$ is the tensile elongation of the H-charged tensile test specimens. The experiments were repeated at least twice to ensure accuracy.

The hydrogen content was measured by thermal desorption mass spectrometry (TDS), utilizing a G4 PHOENIX DH instrument (Bruker Corporation, Ettlingen, Germany). The samples were measured at 800 °C, with a holding time of 15 min, to force the hydrogen to completely escape during the test. The sample size used for measuring hydrogen content was 15 mm × 1.2 mm × 4.2 mm, and the experimental test was repeated at least twice to ensure the accuracy of the data.

Following pre-straining, the alloy samples were prepared for microstructural and fractographic analysis. The stressed surface layer was first removed by electropolishing in a mixed solution of 10% perchloric acid in ethanol, kept at 273 K with an applied voltage of 25 V. Subsequently, the microstructure was characterized by EBSD, and the fracture morphology, along with hydrogen-induced crack characteristics, was analyzed using SEM.

## 3. Results

### 3.1. Microstructure Characterization

**Figure 1:**
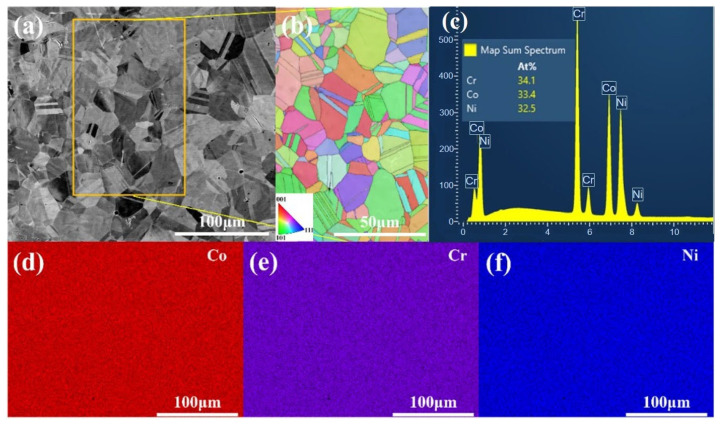
Microstructure and composition of the recrystallized CoCrNi medium-entropy alloy: (**a**) Back scattered electron (BSE) image of the CoCrNi alloy without pre-strain; (**b**) Inverse pole figure (IPF) image of CoCrNi alloy without pre-strain; (**c**) Quantitative results of elemental composition of the CoCrNi alloy without pre-strain, and the elemental distribution results of (**d**) Co; (**e**) Cr; and (**f**) Ni.

Figure 1a, a backscattered electron (BSE) image of the recrystallized CoCrNi MEA without pre-strain, shows a uniform microstructure. The lack of atomic number-related contrast variation indicates an absence of compositional segregation or secondary phases, confirming the high homogeneity of the alloy. An inverse pole figure (IPF) map (corresponding to the yellow frame region in Figure 1a), shows randomly oriented equiaxed grains with an average size of 16.40 μm (Figure 1b). The uniform color distribution indicates no pronounced texture, confirming complete recrystallization and a homogeneous grain structure. Figure 1c shows the quantitative analysis results of elemental composition obtained by energy dispersive spectrometer (EDS) mapping. The Cr content was 34.1 at%, Co content was 33.4 at%, and Ni content was 32.5 at%. Figure 1d–f display uniform spatial distributions of Co, Cr, and Ni without segregation or precipitation, confirming excellent compositional homogeneity across scales. This uniformity aligns with medium-/high-entropy alloy characteristics and ensures consistent mechanical properties.

**Figure 2:**
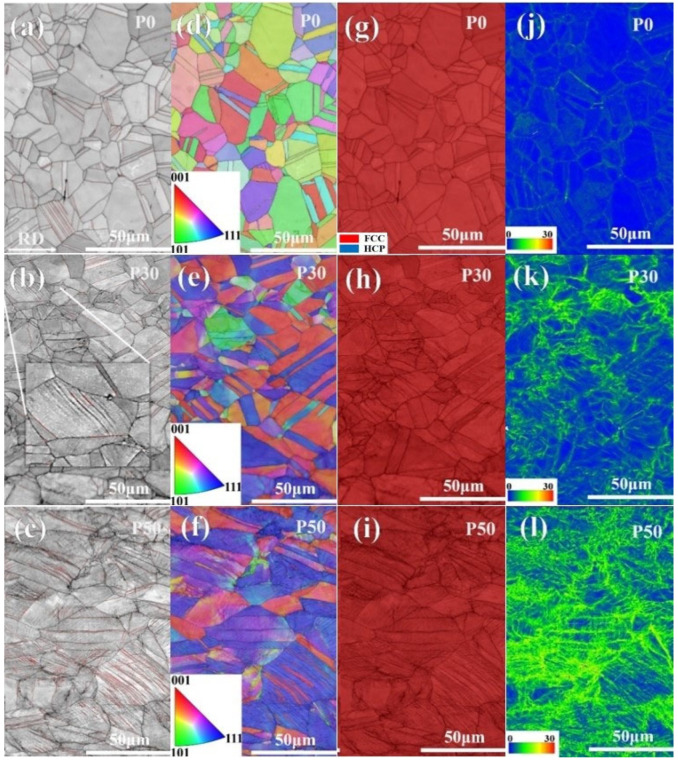
Evolution of microstructure and geometrically necessary dislocation (GND) density with pre-strain: (**a**–**c**) Kikuchi pattern qualities (KPQ) images of the P0, P30, and P50 samples (**a**–**c**), respectively, where the red lines indicate twin boundaries; (**d**–**f**) EBSD-IPF images of the P0, P30, and P50 samples, respectively; (**g**–**i**) Phase distribution images of the P0, P30, and P50 samples, respectively; (**j**–**l**) GND images profiles of the P0, P30, and P50 samples, respectively, where the unit of GND was 10^14^ m^−2^.

Figure 2 illustrates the distinct microstructures after pre-strain. Figure 2a,d display the microstructure of P0 sample, which primarily consists of equiaxed grains and annealing twins, with no texture formation. The rolling direction (RD), indicated in Figure 2a, also corresponds to the tensile testing direction. Figure 2b,e present the microstructure of P30 sample, showing significant changes in grain orientation (Figure 2e) and elongation of grains along the deformation direction. Additionally, a small number of deformation twins are observed within the grains of the P30 sample. Unlike coarse annealing twins, the deformation twins exhibit finer twin thickness and smaller twin spacing, as shown in Figure 2b. Figure 2c,f show the microstructure of the P50 sample. Similarly to the P30 sample, notable changes in grain orientation occur, accompanied by a higher density of deformation twins, as marked by the red lines at twin boundaries in Figure 2c. Thus, these deformation twins play a critical role in the plastic deformation of the CoCrNi MEA, consistent with previous studies [26,27].

Figure 2g–i show the phase structure and distribution of geometrically necessary dislocation (GND) densities in the three specimens. Figure 2g–i indicate that all specimens maintained a stable FCC structure during pre-straining, with no martensitic phase transformation occurring. As shown in Figure 2j–l, the GND density in the samples increased with engineering strain. Statistical results reveal GND densities of 0.51 × 10^14^ m^−2^ for P0 sample, 4.88 × 10^14^ m^−2^ for P30, and 8.16 × 10^14^ m^−2^ for P50. In P0 and P30 samples, GNDs were primarily distributed along GBs, with P30 sample exhibiting higher GND density than P0 sample. In contrast, P50 sample showed high GND density both at GBs and within grains due to the presence of extensive deformation twin boundaries. This phenomenon can be attributed to the suitable thickness of deformation twins and the accumulation of immobile dislocations, which enhance the material’s strength [28].

### 3.2. Mechanical Properties

**Figure 3:**
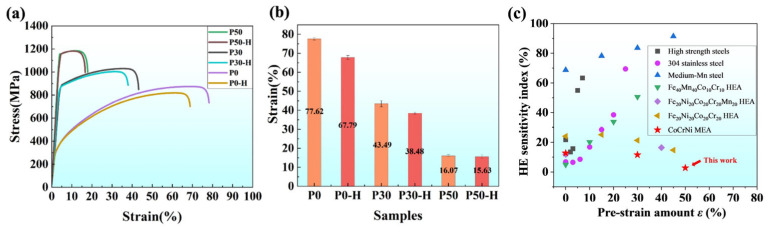
Mechanical properties of all original and pre-charged hydrogen samples: (**a**) Engineering stress–strain curves of P0, P30, and P50 and their H-charged samples; (**b**) Elongation of P0, P30, and P50 and their H-charged samples; (**c**) A comparison of hydrogen embrittlement resistance data among equiatomic CoCrNi MEA, traditional alloys, and currently reported HEAs.

The mechanical properties of all original and pre-strained hydrogen-charged samples were tested by slow strain rate tensile testing, with the results shown in Figure 3a,b. The P0 sample exhibited high elongation (77.62 ± 0.67%) and low yield strength (293.79 ± 5.44 MPa). Through pre-straining, both the yield strength and tensile strength of P30 and P50 were improved. P30 was strengthened primarily by the introduction of a high density of dislocations, while P50 was enhanced by the combined effects of dislocations and a considerable number of deformation twins. The yield strength of P30 was 882.14 ± 6.24 MPa, and that of P50 was 1163.88 ± 4.40 MPa. The elongation was 43.49 ± 1.68% for P30 and 16.07 ± 0.54% for P50. Hydrogen charging mainly influenced the plastic properties of the CoCrNi MEA. Based on the elongation data in Figure 3b, the elongation loss (*EI*_loss_) was 12.67% for P0, 11.51% for P30, and 2.74% for P50. While the *EI*_loss_ values of P0 and P30 were similar, while a sharp decrease of *EI*_loss_ was observed in P50, indicating significantly improved HE resistance. which is closely associated with the presence of deformation nano-twins in its microstructure and their interaction with hydrogen. Figure 3c presents a comparison of HE susceptibility indices (e.g., elongation loss) between our CoCrNi medium-entropy alloy samples and reported data for high-strength steels [29], stainless steels [17], medium-Mn steels [30], and other high-entropy alloys (HEAs) [31,32,33]. It can be observed that the nano-twinning structure strategy introduced in our best-performing P50 sample achieves a superior combination of strength and HE resistance.

### 3.3. Fracture Surface and Gauge Surface Analysis

**Figure 4:**
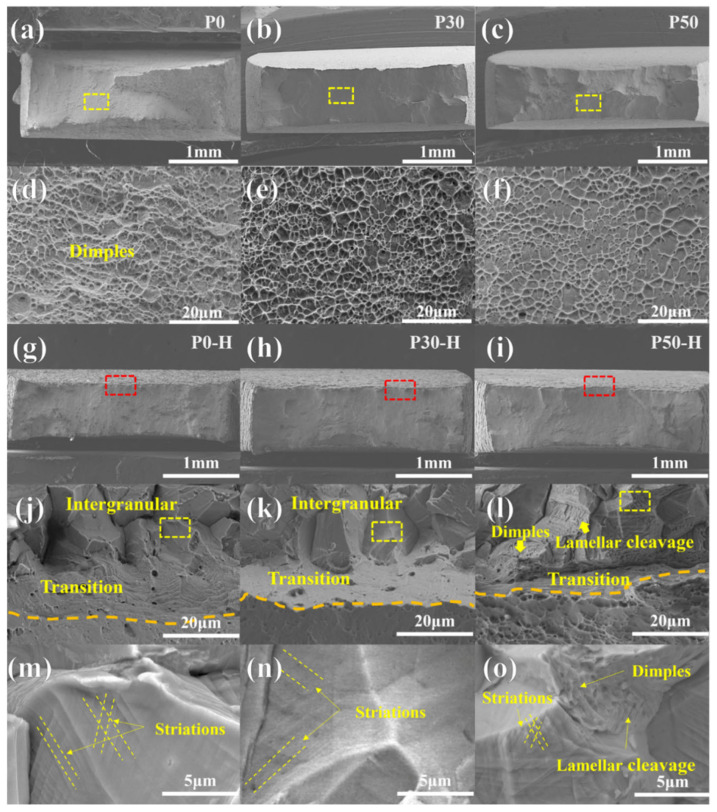
Microstructural fracture morphology of tensile specimens without and with hydrogen charging: (**a**) P0; (**b**) P30; and (**c**) P50; (**d**–**f**) Local amplification images of the yellow dotted square areas in (**a**–**c**); (**g**) P0-H; (**h**) P30-H; (**i**) P50-H; (**j**–**l**) Local amplification images of the red dotted square areas in (**g**–**i**); (**m**–**o**) Local amplification images of the yellow dotted square region in (**j**–**l**), where the orange dotted lines in (**j**–**l**) are ductile fracture and brittle fracture bound.

Figure 4 presents the fracture morphologies of the different samples after tensile testing. Under hydrogen-free conditions, the fracture surfaces of all samples were characterized by dense dimples. With increasing engineering strain levels, the dimple size on the fracture surfaces gradually decreased and became shallower, as shown in Figure 4d–f. This phenomenon may be attributed to the variation in elongation among the samples. Generally, large and deep dimples are observed in materials exhibiting higher elongation. As demonstrated in Figure 4a–c, the cross-sectional shrinkage decreased progressively with the degree of engineering strain. In summary, the fracture mode of the P0, P30, and P50 samples in the absence of hydrogen charging was ductile fracture.

Figure 4g–o present the fracture morphologies of hydrogen-charged samples after slow strain rate tensile testing. As shown in Figure 4g–i, a brittle zone appeared in the peripheral region of the fracture surface, primarily caused by the slow diffusion rate of hydrogen. For instance, although electrochemical hydrogen charging could not fully permeate the single-phase FCC structured alloy [12,16,34], the fracture edge exhibited a brittle zone while the central region still contained numerous dimples. Figure 4j–l display the fracture surface morphologies of P0-H, P30-H, and P50-H, respectively. Figure 4j shows that P0-H contained a brittle zone characterized by intergranular cracking and a transition zone where the fracture mode was between ductile and brittle fracture. At higher magnification (Figure 4m), smooth intergranular fracture planes with parallel striation patterns were observed. These striations represent local deformation features induced by HE [35,36]. Figure 4k indicates that P30-H exhibited a similar distribution of brittle, transition, and ductile zones as shown in Figure 4j, with the brittle zone still dominated by intergranular cracking. Figure 4n reveals striations indicative of local deformation within the brittle zone of the P30-H sample. In contrast to the typical intergranular brittle fracture morphology observed in the previous two samples, the hydrogen-affected region of the P50-H sample exhibited a mixed fracture mode, including fine dimples and lamellar cleavage morphology (Figure 4l,o). Although intergranular cracking and local deformation striations were also present, these striations were coarser, suggesting better plasticity. Furthermore, the fracture surface of P50-H was less smooth than those of P0-H and P30-H, indicating retained ductility under hydrogen influence [36]. In summary, the introduction of deformation twins in the P50 and P50-H samples altered the hydrogen-affected fracture mode.

**Figure 5:**
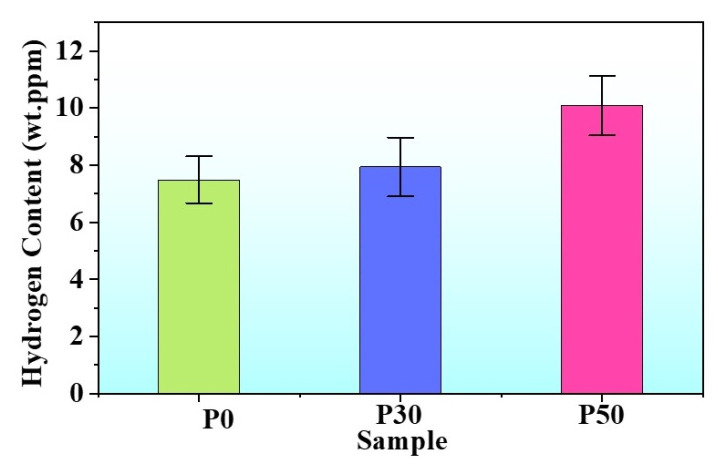
Hydrogen content of the samples after 48 h of hydrogen charging.

Figure 5 shows the variation in hydrogen content with pre-strain level. As the engineering strain increased, the hydrogen content gradually rose. The internal hydrogen contents of the P0, P30, and P50 samples were approximately 7.49 ppm, 7.94 ppm, and 10.10 ppm, respectively. This increase in hydrogen content is attributed to dislocations, deformation twins, and hydrogen traps within the samples, which can capture additional hydrogen. These hydrogen traps reduce the diffusivity of hydrogen in the alloy [22,37]. As shown in Figure 4j–l, the average depths of the hydrogen-affected zones for the P0, P30, and P50 samples were 34.1 ± 0.7 μm, 33.9 ± 0.8 μm, and 27.8 ± 1.7 μm, respectively, indicating a decreasing trend in the depth of the brittle zone.

**Figure 6:**
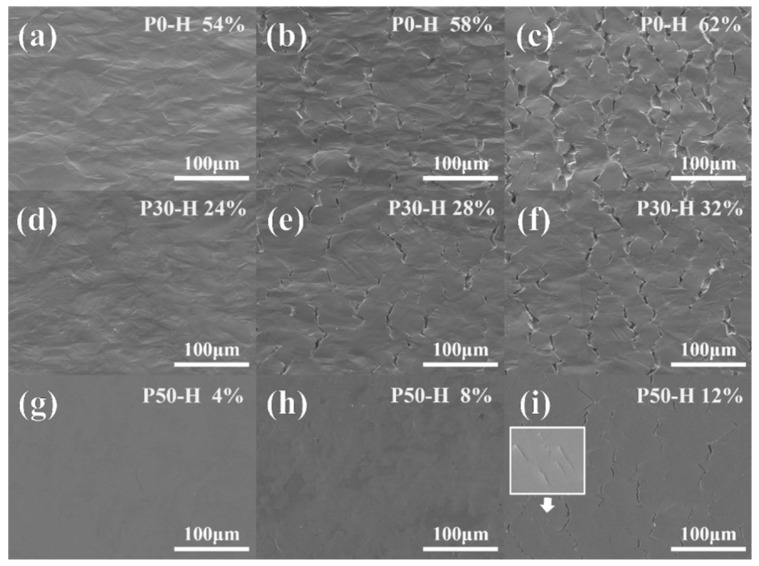
Crack morphology of the all samples after H-charging: (**a**) P0-H was stretched to 54% engineering strain; (**b**) 58% engineering strain; and (**c**) 62% engineering strain; (**d**) P30-H was stretched to 24% engineering strain; (**e**) 28% engineering strain; and (**f**) 32% engineering strain; (**g**) P50-H was stretched to 4% engineering strain; (**h**) 8% engineering strain; and (**i**) 12% engineering strain.

Furthermore, crack nucleation and propagation characteristics of the hydrogen-charged samples were analyzed in detail using SEM, as shown in Figure 6. Three different engineering strain levels were applied to each sample, with the total strain level kept consistent across all three samples at each strain stage. The total strain level is defined as the sum of pre-strain and post-hydrogen-charging tensile strain. For example, as compared to the undeformed sample, the total strain level shown in Figure 6a,d,g was uniformly 54%. The total strain levels were set into three groups: 54%, 58%, and 62%. At the total strain level of 54%, few cracks were initiated in all samples, and the cracks in all three samples were at the nucleation stage.

**Figure 7:**
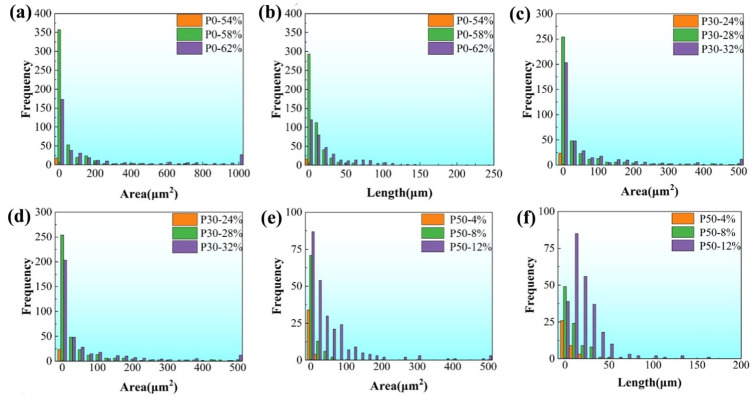
The distribution of crack area and length frequency for all hydrogen-charged samples under varying applied strain levels: (**a**,**b**) Frequency distributions of the crack area and length of the P0 sample under different strain levels; (**c**,**d**) Frequency distribution of the crack area and length of P30 under different strain levels; (**e**,**f**) Frequency distribution of the crack area and length of P50 under different strain levels.

For the hydrogen-charged tensile specimen, an area measuring 400 μm × 300 μm at the center of the gauge length was selected for a detailed analysis of crack morphology and associated characteristics. Figure 7 comprehensively illustrates the frequency distribution of both crack area and length under varying applied strain levels. As clearly depicted in Figure 7a,b, the initiation and subsequent propagation of cracks occur with remarkable rapidity as the total strain level is elevated from 54% to 58%. This characteristic is similarly evident in Figure 7c,d, wherein the early-stage crack propagation behaviors of specimens P0 and P30 are observed to be qualitatively comparable. A critical transition is noted when the total strain level progresses from 58% to 62%. Within this range, the population of small-scale cracks, which are defined as having an area less than 100 μm^2^ and a length under 50 μm, undergoes a pronounced decline in specimen P0. In stark contrast, the quantity of such small-sized cracks in specimen P30 remains largely unchanged, showing no significant reduction. Notably, as shown in Figure 7a, the attainment of a 62% strain level in the P0 specimen leads to the emergence of exceptionally large cracks possessing areas greater than 1000 μm^2^. The formation of these super-sized cracks is attributed to the synergistic mechanisms of vigorous crack propagation and active coalescence (merging) of smaller cracks, a process which consequently depletes the population of smaller cracks. This progression is further substantiated by a direct comparison between Figure 6b,c, the latter revealing a substantial and evident increase in both the average crack length and area. Conversely, Figure 7e,f reveal a distinctly different trend in the crack evolution for specimen P50. The primary characteristic observed during the strain increase from 54% to 58% is the nucleation of a high density of small-scale cracks. It is only upon reaching the higher strain level of 62% that these cracks undergo significant expansion. In comparison to the behaviors documented for P0 and P30, the entire processes of crack initiation and propagation in specimen P50 are markedly delayed. This retardation effect is likely attributable to an alteration in the hydrogen distribution within the P50 material, which consequently mitigates the severity of local stress concentrations. These localized stress concentrations are recognized as critical sites for crack nucleation and subsequent growth.

**Figure 8:**
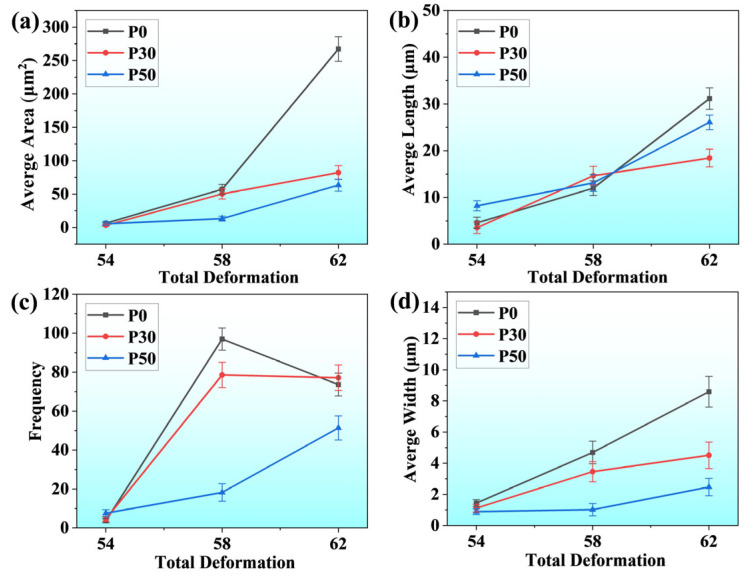
Crack statistics of different samples at 54%, 58%, and 62% total strain level: (**a**) Average crack area at different total deformation levels; (**b**) Average crack length at different total strains; (**c**) Average number of cracks at the total deformation level; (**d**) Average crack width at different total deformation levels.

Figure 8 provides a comprehensive statistical overview of crack morphological features across different specimens subjected to varying total strain levels, with the analyzed crack population exceeding 1800 individual instances. As illustrated in Figure 8a, a continuous upward trend in average crack area is observed with increasing total strain. Similarly, Figure 8b demonstrates a correlated increase in average crack length as the overall strain level escalates. With regard to crack quantity, even under conditions of simultaneous crack initiation, the P50 consistently exhibits a comparatively lower number of cracks, as clearly depicted in Figure 8c. A particularly noteworthy phenomenon occurs in the P0 when total strain rises from 58% to 62%, where a dramatic reduction in crack count is observed. This phenomenon is primarily attributed to two synergistic mechanisms: extensive crack propagation along the tensile direction and the progressive coalescence of numerous fine-scale micro-cracks. To quantitatively evaluate the degree of crack extension along the loading axis, this study implemented a simplified yet effective methodological approach by calculating the ratio of crack area to crack length, thereby deriving an estimated crack width parameter. The corresponding statistical results are systematically presented in Figure 8d. Detailed analysis reveals that during the critical strain transition from 58% to 62%, the P0 maintains a consistently high crack propagation rate. In contrast, the P30 exhibits a discernible deceleration in propagation kinetics compared to its performance during the 54–58% strain interval. The P50 demonstrates particularly interesting behavior, maintaining essentially stable crack widths throughout the 54–58% strain range, with significant width expansion only manifesting when strain reaches the 58–62% threshold. Remarkably, even at the maximum investigated strain level of 62%, the P50 retains crack widths at a relatively modest level. The crack evolution patterns documented in Figure 7 and Figure 8 exhibit complete consistency with the microscopic observations presented in Figure 6, thereby validating the methodological approach and analytical conclusions. In summary, under equivalent total strain, cracks in P0 and P30 specimens exhibit markedly greater extension along the tensile direction and an earlier transition to unstable growth than those in P50. These distinct propagation behaviors elucidate the material’s strain accommodation and damage tolerance mechanisms.

## 4. Discussion

In this study, the P0, P30, and P50 specimens correspond to three distinct microstructural states: dislocation-free and deformation twin-free, containing a high density of dislocations with a small number of deformation twins, and possessing a substantial population of both deformation twins and dislocations. This can be attributed to the evolution of deformation mechanisms in CoCrNi MEAs with increasing strain level and time. The initial deformation is primarily characterized by typical dislocation structures of low stacking fault energy FCC alloys, resulting in the generation of a high density of dislocations. Upon reaching a critical stress level at later stages, deformation twins (i.e., nano-twin structures) are formed [15,26]. These pre-strain-induced variations in internal deformation structures significantly influence hydrogen transport and distribution. In the P0 specimen, deformation prior to crack initiation occurs mainly through planar dislocation glide, while hydrogen is transported by mobile dislocations [38]. The continuous interaction between dislocations and GBs alters the atomic configuration at GBs [39], introducing more defects. This facilitates enhanced hydrogen transport to GBs and creates high free volume for hydrogen trapping [40], leading to severe hydrogen enrichment at grain boundaries. The primary fracture mode in this specimen is intergranular fracture, characterized by smooth cleavage facets (Figure 4j,m). This morphological feature, combined with the severe hydrogen enrichment at grain boundaries resulting from dislocation-mediated transport, strongly indicates the dominance of the HEDE mechanism. Under such low-strength and high-ductility conditions, hydrogen accumulation at grain boundaries is sufficient to weaken interatomic cohesion, leading to brittle fracture before significant local plastic deformation occurs.

Studies have shown that twin boundaries exhibit minimal free volume for hydrogen trapping and higher hydrogen dissolution energy, making them less favorable for hydrogen absorption and transport [41]. The CoCrNi alloy, with its low stacking fault energy (22 mJ m^−2^), facilitates the formation of nanoscale deformation twins during deformation [42,43,44]. This reduces the number of dislocations that can be accommodated within the matrix and increases the stress required for dislocations to cross twin boundaries. Since the generated dislocations are primarily non-screw type, they dissociate into partial dislocations upon encountering deformation twin boundaries, with Shockley partials remaining at the twin boundaries [28,45]. Within the same strain range, the pre-existing twins in the P50 specimen effectively hinder dislocation motion, thereby suppressing dislocation-mediated hydrogen transport and reducing hydrogen accumulation at GBs. The presence of deformation twins also promotes a more homogeneous distribution of hydrogen. Consequently, this mitigates stress concentration induced by the interaction between newly formed deformation twin boundaries and GBs during subsequent deformation. The enhanced HE resistance of the P50 specimen can be attributed to the suppression of conditions favorable for HEDE. However, the residual ductility loss observed even in P50 indicates that other mechanisms remain active. The high density of pre-existing deformation twins not only reduces hydrogen transport to grain boundaries but also strongly impedes dislocation motion. This impediment likely suppresses the hydrogen-enhanced localized plasticity (HELP) mechanism, which relies on hydrogen facilitating dislocation mobility and multiplication in localized regions [46]. Therefore, in the P50 specimen, the primary role of the nano-twins is to mitigate the key driver for HEDE (localized supersaturation) while simultaneously restricting the plasticity localization central to HELP. Compared to the P0 specimen, the pre-existing deformation twins in the P30 specimen partially obstruct planar dislocation glide and reduce the extent of dislocation-assisted hydrogen transport. For the P30 specimen, a synergistic interaction of both mechanisms is probable. The partial obstruction of dislocation glide by a limited number of twins reduces, but does not eliminate, severe hydrogen segregation at grain boundaries, allowing HEDE to initiate microcracks. Subsequently, these microcracks may propagate in a manner assisted by the HELP mechanism in the surrounding matrix. This transition from HEDE-dominated initiation to HELP-assisted propagation is a recognized failure pathway in many alloys. Overall, the pre-introduced high density of nano-twins in the P50 specimen significantly impedes hydrogen transport, homogenizes hydrogen distribution, and attenuates the degradation of GB cohesion. The strategic value of this work lies in its microstructural design, which successfully transforms the material’s HE failure mode from HEDE-dominated behavior to co-suppression of both HEDE and HELP mechanisms, thereby effectively enhancing the resistance to HE. This approach provides a practical materials science solution to the fundamental challenge of decoupling mechanistic contributions in HE research.

The HE susceptibility of the pre-strained CoCrNi alloy was evaluated by electrochemical charging and tensile testing. Although this accelerated method differs from actual service conditions due to the introduction of excessively high hydrogen concentrations, the hydrogen-microstructure interaction mechanisms it reveals are universally applicable. We critically demonstrate that pre-existing deformation twins significantly enhance HE resistance by homogenizing hydrogen distribution and impeding dislocation-assisted transport, establishing a robust microstructural criterion for designing HE-resistant alloys. Future work should validate these findings under real service conditions through long-term exposure and slow strain rate testing to quantitatively assess the alloy’s engineering application potential.

## 5. Conclusions

P0 exhibited a fully recrystallized microstructure containing annealing twins. With increasing pre-strain levels, the dislocation density rose substantially, accompanied by the formation of nanoscale deformation twins. Pre-strain enhanced the alloy’s strength at the expense of ductility. The P50 sample achieved the highest yield strength (1163.88 MPa) due to composite strengthening from both dislocations and twins. After hydrogen charging, the elongation losses were 12.67% for P0, 11.51% for P30, and only 2.74% for P50, demonstrating that pre-introduced deformation twins significantly improved the alloy’s HE resistance. Uncharged specimens all exhibited ductile fracture characterized by dimples. Following hydrogen charging, P0 and P30 samples developed intergranular brittle fracture zones with smooth cleavage planes and striations, indicating dominance of the HEDE mechanism. In contrast, P50 showed a mixed fracture mode with finer dimples and retained plasticity, suggesting that twins alleviated hydrogen-induced embrittlement. Hydrogen content increased with pre-strain due to trapping at dislocations and twins. Under identical strain levels, P50 demonstrated delayed crack initiation, slower propagation rates, and fewer large cracks compared to P0 and P30. This is attributed to the pre-existing twins, which impeded dislocation glide and hydrogen transport, promoted uniform hydrogen distribution, alleviated grain boundary stress concentration, and thereby suppressed crack coalescence and unstable growth.

## Data Availability

The original contributions presented in the study are included in the article, further inquiries can be directed to the corresponding author.
